# Severe intraprosthetic aortic insufficiency immediately after implantation of a SAPIEN 3 Ultra RESILIA valve with spontaneous resolution: case report

**DOI:** 10.1093/ehjcr/ytae402

**Published:** 2024-08-06

**Authors:** Juan Armando Diaz, Ken Kobayashi, Daisuke Hachinohe, Ryo Horita, Hidemasa Shitan

**Affiliations:** Cardiovascular Medicine, Asia Medical Group, Sapporo Heart Center, Sapporo Cardio Vascular Clinic, North 49, East 16, 8-1, Higashi Ward, Sapporo 007-0849, Japan; Makati Medical Center, Makati, Philippines; Cardiovascular Medicine, Asia Medical Group, Sapporo Heart Center, Sapporo Cardio Vascular Clinic, North 49, East 16, 8-1, Higashi Ward, Sapporo 007-0849, Japan; Cardiovascular Medicine, Asia Medical Group, Sapporo Heart Center, Sapporo Cardio Vascular Clinic, North 49, East 16, 8-1, Higashi Ward, Sapporo 007-0849, Japan; Cardiovascular Medicine, Asia Medical Group, Sapporo Heart Center, Sapporo Cardio Vascular Clinic, North 49, East 16, 8-1, Higashi Ward, Sapporo 007-0849, Japan; Cardiovascular Medicine, Asia Medical Group, Sapporo Heart Center, Sapporo Cardio Vascular Clinic, North 49, East 16, 8-1, Higashi Ward, Sapporo 007-0849, Japan

**Keywords:** Aortic valve stenosis, Complication, Intravalvular aortic insufficiency, Intraprosthetic aortic insufficiency, Transcatheter aortic valve replacement, Case report

## Abstract

**Background:**

Aortic insufficiency (AI) is often encountered in transcatheter aortic valve replacement (TAVR) but is only rarely haemodynamically significant. Even more uncommon is the occurrence of intraprosthetic AI that often leads to haemodynamic compromise requiring additional therapeutic intervention.

**Case summary:**

An 85-year-old female with severe aortic stenosis underwent elective TAVR with a size 23 mm SAPIEN 3 Ultra RESILIA (S3UR) valve. After implantation, the patient developed hypotension. Transthoracic as well as transoesophageal echocardiogram identified significant transvalvular AI. Persistence of AI led the team to consider a TAV-in-TAV strategy. Before a second valve system could be inserted, the patient’s blood pressure improved and AI resolved spontaneously. The patient was discharged and recovered with no AI on follow-up imaging studies.

**Discussion:**

This is the first reported case of significant intraprosthetic AI with the new S3UR in our literature search as of writing. The new S3UR valve has several improvements from previous generations designed to increase valve area and durability as well as decreasing the incidence of paravalvular and intraprosthetic leaks. A stuck leaflet was inferred as the cause of the intraprosthetic AI, and the improved design with excessive expansion may have led to this. Unlike previous case reports, intraprosthetic AI resolved without further intervention likely due to the turbulence of flow releasing the stuck leaflet. Cautious observation prevented the need for a TAV-in-TAV for a rare and possibly catastrophic complication.

**Learning points**

- Intraprosthetic aortic insufficiency is rare and particularly life-threatening, which may occur despite the latest technology and innovation thus requiring constant vigilance.
- The approach to intraprosthetic aortic insufficiency should be individualized considering the patient’s clinical condition, imaging, and haemodynamic information.

## Introduction

Intravalvular or transvalvular aortic insufficiency is a complication of transcatheter aortic valve (TAV) replacement that is not often encountered. It is often attributed to leaflet restriction, destruction, or incorrect sizing of the valve.^1^ Transvalvular aortic insufficiency (AI) has often required mechanical manoeuvres or a valve-in-valve strategy to resolve.^2–5^ We report a case of severe intraprosthetic aortic insufficiency immediately after implantation of a transcatheter heart valve with spontaneous resolution.

## Summary figure

**Figure:**
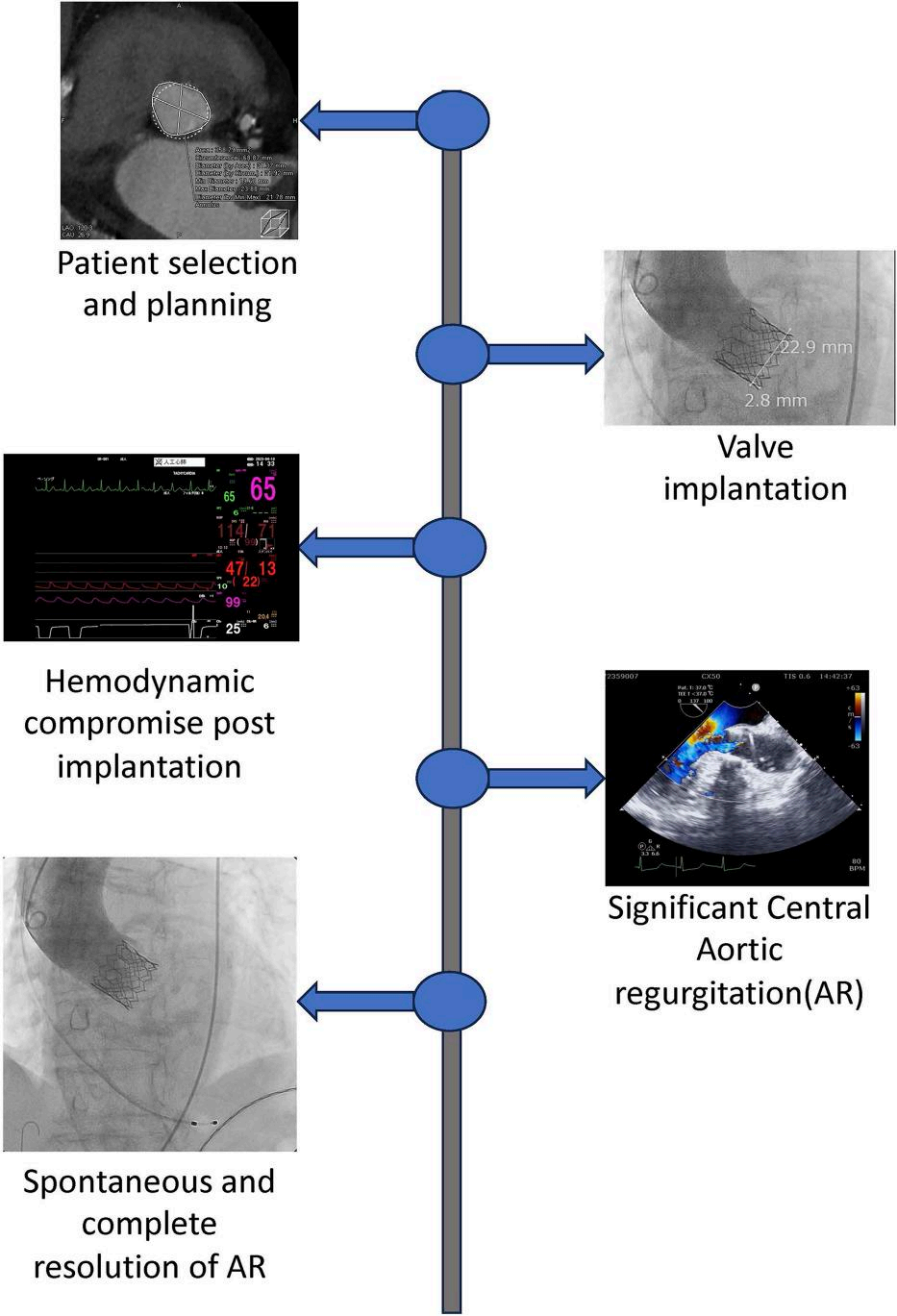


## Case presentation

**Figure 1:**
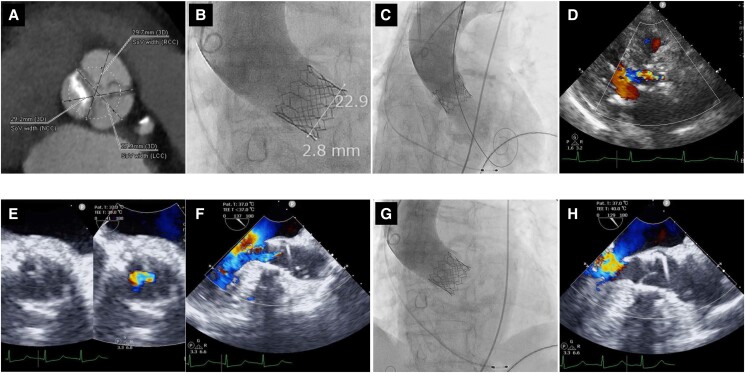
(*A*) Dense calcification at the non-coronary cups. (*B*) Final implantation position of valve. (*C*) Angiography and (*D*) transthoracic echocardiography demonstrating significant aortic insufficiency (AI). (*E*, *F*) Significant, central, transvalvular AI without prolapse by transoesophageal echocardiography (TEE). (*G*) Final angiography showing no AI. (*H*) Final TEE showing no AI.

**Figure 2:**
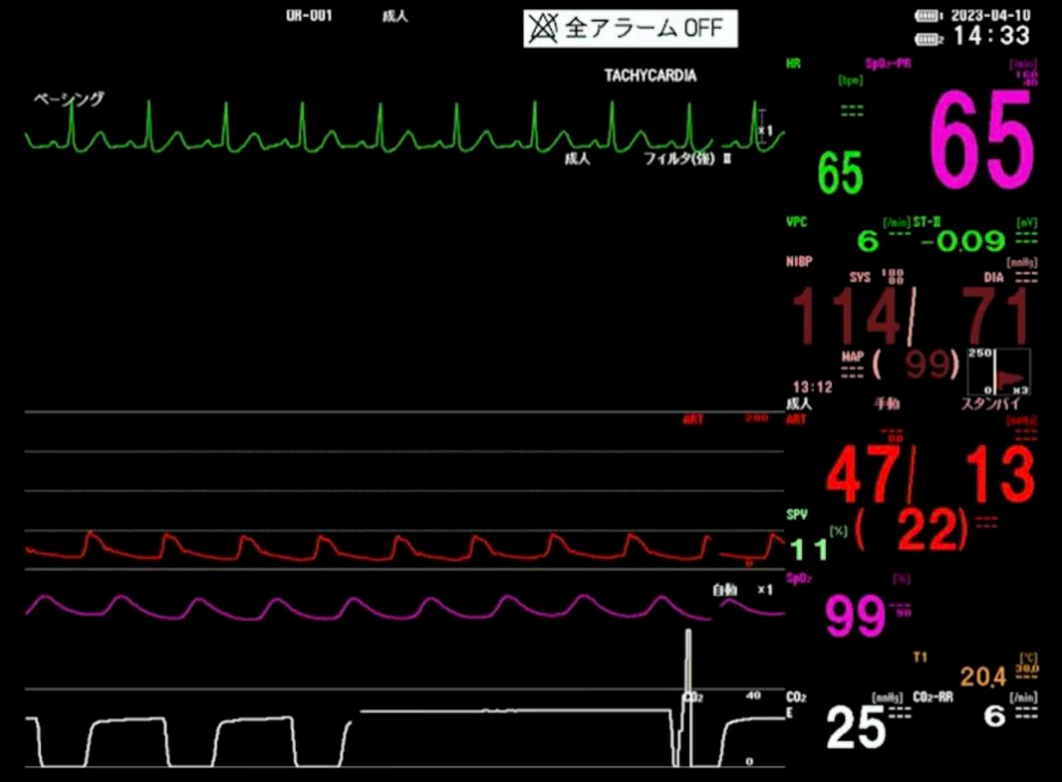
Patient haemodynamics during AI.

An 85-year-old female with symptomatic severe aortic stenosis and end-stage renal disease on haemodialysis had a recent pelvic fracture and prior to orthopaedic surgery, discussed the option of treatment for aortic stenosis. Her pre-operative vital signs were stable with a blood pressure of 120/60 mmHg, heart rate of 74 beats per minute, and no desaturations. Transthoracic echocardiography (TTE) at this time showed an aortic valve area of 0.83 cm^2^ by continuity equation with a peak velocity of 4.1 m/s, a mean pressure gradient of 40 mmHg, and no aortic regurgitation. Left ventricular function was normal with an ejection fraction of 69%, and the right ventricle had normal dimensions and good contractility. Due to high frailty, the heart team recommended TAV replacement. Pre-operative planning through computed tomography measurements was done revealing an aortic annular area of 359 mm^2^ and no left ventricular outflow tract calcification. Measurements from the left ventricular outflow tract, sinuses of Valsalva, and sinotubular junction were also considered adequate. Right subclavian artery approach via cutdown under general anaesthesia with endotracheal intubation was selected with a SAPIEN 3 Ultra RESILIA (S3UR, Edwards Lifesciences, Irvine, CA, USA) size 23 mm valve. The size 23 mm valve would be ∼13% oversized at nominal inflation (406 mm^2^). Right ventricular temporary pacing was done through the left femoral vein. Access and delivery were unremarkable and without significant resistance. The valve was deployed without predilatation, at −1 cc underfilled and positioned at approximately the −2.8 mm position with rapid pacing at 180 b.p.m. (*Figure 1B*). The patient remained hypotensive with blood pressure of 47/13 mmHg, and TTE and angiography showed severe transvalvular AI (*Figure 1C* and *D*, *Figure 2*). Contractility remained adequate, mitral regurgitation was mild, and there were no signs of aortic rupture nor coronary occlusion. Norepinephrine was started to improve the blood pressure. Delivery system, left ventricular wire, and the pigtail catheter were removed but AI remained significant on TTE (see Supplementary material online, *Videos*). Persistent significant transvalvular AI was confirmed by transoesophageal echocardiography (TEE) without paravalvular leak (*Figure 1E* and *F*). The decision was made to pursue a TAV-in-TAV strategy to treat the significant AI. However, AI was noted to resolve spontaneously by TEE and confirmed by angiography (*Figure 1G* and *H*). The procedure was concluded with surgical vascular closure of the subclavian access site and the patient was discharged after an uneventful post-operative course and no AI on follow-up echocardiograms as outpatient.

## Discussion

This is the first reported case of severe transvalvular AI after implantation of the new S3UR transcatheter heart valve (THV). Aortic insufficiency may be caused by leaflet dysfunction due to sizing, implanting, or mounting. Transvalvular AI, however, is a rare complication for THV and in our review, there have only been seven cases of transvalvular aortic regurgitation after implantation of a THV reported since 2010.^2–8^ Previous cases have required prompt decision making and rapid intervention, ranging from balloon inflation to valve-in-valve strategies. Current guidelines make no specific mention of transvalvular and intraprosthetic aortic insufficiency but do cite transcatheter closure or reoperation strategies as viable options for patients with significant paravalvular leaks, with the choice of intervention determined by the cause.^9^

**Figure 3:**
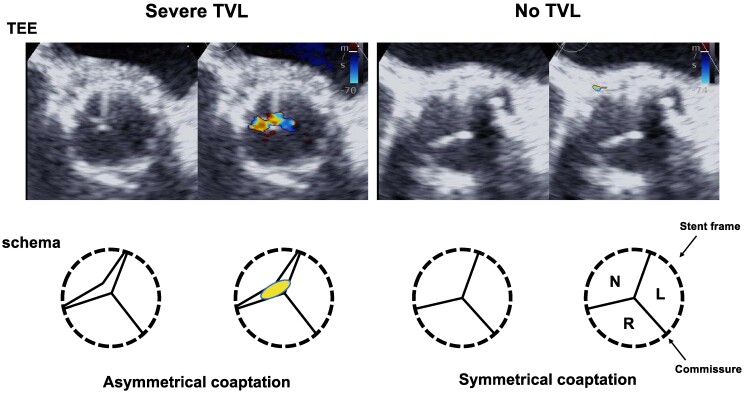
TEE short axis view of leaflet coaptation with and without AI.

The latest generation of THV, the S3UR, boasts of a new frame, new leaflets, and a new skirt, all designed to decrease paravalvular leaks and prevent leaflet malcoaptation, interference of leaflets by the frame, and transvalvular leaks.^10^ The new design also decreases strut foreshortening and allows for an improved fit across sizes. This improved fit includes an increased effective orifice area, particularly for the 20 and 23 mm valve sizes. Previous reports have cited stuck or damaged leaflets as the cause of transvalvular insufficiency requiring mechanical dislodgement to release the stuck leaflet, such as the use of the pigtail catheter or reinflation of an overfilled balloon.^2,4,11^ Overdilatation of the valve in larger annular sizes has likewise been mentioned as a predictor for transvalvular insufficiency due to central leaflet separation.^1^ In this case, we infer that the skirt was conversely inflated with an underfilled balloon. Inadequate expansion may have led to entrapment of the leaflet to the frame in a deformed state, as demonstrated by asymmetrical coaptation thus resulting in transvalvular insufficiency (*Figure 3*, Supplementary material online, *Videos*). An *in vitro* study of the SAPIEN XT 23 mm valve showed leaflet distortion and a prolonged valve closing time.^12^ The effects in that paper, however, were not haemodynamically significant unless underfilling was less by 3 cc.^12^ Another study that used data from the OCEAN-TAVI registry concluded that underfilling or overfilling the SAPIEN XT was safe, but found that the size 23 mm valve had a propensity to a higher transvalvular gradient.^13^ This case used a 23 mm SAPIEN Ultra RESILIA, a more advanced version of the THV but may still be susceptible to similar effects of underfilling. Impingement of the leaflets by the wire or pigtail was not considered due to the shape and location of the AI as well as its persistence despite the removal of all devices. Fortunately, haemodynamic compromise was not severe thus providing time for observation and allowing the turbulence from the AI to gradually release the trapped leaflet leading to a normally functioning valve. This case, unlike those previously reported, minimized the temporal and financial costs of the procedure as well as decreasing the risk for further complications by avoiding further intervention.

In conclusion, we present a case of significant transvalvular AI after implantation of a S3UR THV. It is the first case of transvalvular AI reported, based on our research, for the newest edition of the valve and illustrates the importance of continued vigilance despite the advancement of technology and safety of these devices. It also highlights the need to individualize therapeutic decisions with consideration for the patient’s haemodynamics and clinical status suggesting the opportunity for conservative management in similar select cases. Further studies into the effect of underfilling on leaflets and haemodynamics may also be pursued as demonstrated by this case.

## Lead author biography


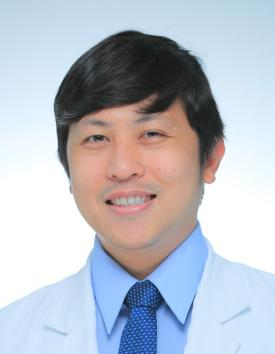


Juan Armando Diaz completed his Medical Degree at the University of the Philippines College of Medicine in 2011 and went on to finish his training in Internal Medicine and Cardiology at the Makati Medical Center, Philippines. He completed his Clinical Research Fellowship in Echocardiography in the same institution before pursuing clinical training in Interventional Cardiology and Transcatheter Aortic Valve replacement at the Sapporo Cardiovascular Clinic, Sapporo, Japan. He is currently a consultant at the Makati Medical Center and heads the Cardiovascular Diagnostics (Heart Station) of the Our Lady of Mount Carmel Medical Center.

## Supplementary Material

ytae402_Supplementary_Data

## Data Availability

The data underlying this article will be shared on reasonable request to the corresponding author.

## Supplementary material

**Consent:** The authors confirm that written consent for submission and publication of this case report including images and associated text has been obtained from the patients in line with COPE guidance.

**Funding:** None declared.
